# A Case of Delirious Mania Treated with Electroconvulsive Therapy

**DOI:** 10.3390/life13071544

**Published:** 2023-07-12

**Authors:** Beniamino Tripodi, Manuel Glauco Carbone, Irene Matarese, Lorenzo Lattanzi, Pierpaolo Medda

**Affiliations:** 1 Department of Mental Health and Addictions, Division of Psychiatry, ASST Crema, Via Largo Ugo Dossena 2, 26013 Crema, CR, Italy; 2 Pisa-School of Experimental and Clinical Psychiatry, University of Pisa, Via Roma 57, 56100 Pisa, PI, Italy; 3 Department of Medicine and Surgery, Division of Psychiatry, University of Insubria, Viale Luigi Borri 57, 21100 Varese, VA, Italy; 4 Department of Surgical, Medical, Molecular and Critical Area Pathology, Clinical and Health Psychology, University of Pisa, Via Roma 57, 56100 Pisa, PI, Italy; 5 Department of Experimental and Clinic Medicine, Section of Psychiatry, University of Pisa, Via Roma 57, 56100 Pisa, PI, Italy

**Keywords:** delirious mania, electroconvulsive therapy, bipolar disorder, somatic treatment, catatonia

## Abstract

(1) Background: Delirious mania is a neuropsychiatric condition characterized by the rapid onset of delirium, psychosis, and mania. Due to the presence of catatonic signs and symptoms, some authors considered this syndrome to be a specific excited catatonia subtype. Usually, delirious mania is responsive to intravenous benzodiazepines (BZDs) or to electroconvulsive therapy (ECT). (2) Methods: In the present study, we describe the case of a 64-year-old woman with a diagnosis of recurrent major depressive disorder. We assessed the severity of the clinical picture and the response to ECT treatment with the Bush–Francis Catatonia Rating Scale (BFCRS). (3) Results: After eleven sessions of ECT, the patient presented a reduced BFCRS total score, with a resolution of the autonomic abnormalities (temperature, respiratory, and heart rate). (4) Conclusions: These data demonstrate how important it is to diagnose this syndrome as soon as possible to set up an effective therapy, avoiding the use of antipsychotic drugs and preventing potentially fatal complications. The initial administration of BZDs IV and the subsequent ECT application, associated with intensive care of life-threatening general medical conditions, guaranteed us a good level of efficacy in obtaining a complete resolution of the clinical picture.

## 1. Introduction

Delirious mania was first described in 1832 by Calmeil [1] as a rapid appearance of delirium, which included manic and psychotic symptoms not determined by organic pathologies, drug toxicity, or any other mental illness. Luther Bell, however, provided the first systematic description of this syndrome. Known also as “Bell’s Mania”, it was defined as an acute neurobehavioral syndrome which alters the state of consciousness and emotional lability, and causes hallucinations, delusions, hyperactivity, and a reduced need for sleep [2]. But the term “delirious mania” was coined by Kraepelin, who considered this peculiar clinical picture to be the most serious form of mania [3]. In recent years, Fink defined delirious mania as the association of delirium, psychosis, and mania [4]. It was also hypothesized that it could be considered a catatonia subtype, keeping in mind the good response to electroconvulsive therapy (ECT) and the frequent association with catatonic symptoms and signs [5]. In the latest classification, which sees the most severe form of malignant catatonia, and the mildest form of non-malignant catatonia (Kahlbaum syndrome) [6], delirious mania is part of the intermediate syndromes along with excited catatonia [7,8] (Table 1).

Delirious mania and excited catatonia are often difficult to distinguish from one another, but this does not affect the outcome of patients since both forms respond to intravenous (IV) benzodiazepines (BZD) and ECT [9,10]. Actually, delirious mania is, however, not in the classification of the Diagnostic and Statistical Manual of Mental Disorders, Fifth Edition (DSM-5) or that of the International Statistical Classification of Diseases and Related Health Problems (ICD-10) [11,12]. Delirious mania occurs with an acute onset and rapid symptomatic progression. It has a variable course with symptoms that can be grouped as follows: delirium, such as disorientation and alterations in sensorium and consciousness; mania, such as excitement, insomnia, psychomotor restlessness, disorganization, and disinhibition; psychosis, such as hallucinations or delusions; and catatonia, such as echophenomena, stereotypies, posturing, rigidity, catalepsy, waxy flexibility, and staring [13].

The pathogenic hypotheses of catatonia remain unclear. This syndrome may present with increased or decreased motor manifestation, suggesting the involvement of different motor pathways and neurotransmitters. Various neuroanatomy and neurophysiology studies have been conducted, suggesting a central role for the inhibitory and excitatory neurotransmission carried by gamma-aminobutyric acid (GABA) and glutamate, through mechanisms that are not fully defined [14,15].

Prior to the introduction of ECT in 1934, the prognosis of patients with an episode of delirious mania was inauspicious and death occurred within hours or days [16]. Moreover, the use of antipsychotics worsens the clinical presentation [17] with the risk of conversion into a malignant catatonic state [18]. The advent of ECT has greatly improved the prognosis of these patients.

In the present paper, we describe the case of a women who suddenly expressed symptoms of delirious mania and was successfully treated with eleven ECT sessions.

## 2. Case Study

### 2.1. History

A 64-year-old woman, retired, with a previous major depressive episode in 1996 for which she had been successfully treated with tricyclic antidepressants (TCAs) (Clomipramine), came to our attention after being transferred from another hospital. In February 2016 she experienced another depressive episode with an associated increase in anxiety levels, which is why she received selective serotonin reuptake inhibitor antidepressants (SSRIs) (Paroxetine) and serotonin–norepinephrine reuptake inhibitor antidepressants (SNRIs) (Venlafaxine) which had a partial clinical benefit. Then, in August 2016, she was admitted, in state of emergency, with a diagnosis of depressive syndrome and was treated with a SNRI (Duloxetine) and BZD (Delorazepam, Triazolam) with a moderate improvement of the affective symptoms. Moreover, due to the occurrence of initial cognitive decline, a head computed axial tomography (CT) without contrast medium was requested. The exam excluded “focal densitometric changes in the supratentorial site”. Due to the persistence of affective and cognitive symptoms, the patient was hospitalized again with a “mixed state” diagnosis and treatment based on SSRI (Citalopram), SNRI (Venlafaxine), and BZD (Alprazolam), and hypnotics (Zolpidem) was set up, without a significant clinical improvement. In addition, in order to ensure the occurrence of vigilance alteration, a magnetic resonance imaging (MRI) scan was performed, which excluded signs of mass effect. Three other hospitalizations followed with multiple combined therapies based on SSRI (Citalopram), atypical antipsychotics (SGAs) (Olanzapine, Paliperidone, Quetiapine, Clotiapine, Asenapine, Risperidone), neuroleptics (FGA) (Haloperidol, Zuclopentixol, Promazine), mood stabilizers (Sodium Valproate, Oxcarbazepine), BZDs (Delorazepam, Diazepam), and hypnotics (Zolpidem). Nevertheless, there was a rapid involution of the cognitive sphere, with disorientation in spatio-temporal parameters and confusion, so pharmacological tapering was performed and a diagnosis of a manic episode with mixed characteristics was made. A second brain MRI without contrast medium excluded, again, space-occupying injuries. The patient was slightly oppositional, confused, disorientated, and agitated, whilst showing a mnesic deficit, identical acceleration, anguished worries, and fragmented and repetitive speech. A therapeutic trial based on Aripiprazole was undertaken, with no response, followed by a second trial with Haloperidol that caused psychic sedation in association with paratonic and catatonic aspects for which it was interrupted. An approach with mood stabilizer drugs (Lithium and Sodium Valproate) and subsequently with BZD IV (Diazepam) was attempted without substantial benefit. Meanwhile, the patient’s clinical picture was progressively worsening, with marked opposition, mutism, staring, and difficulty swallowing (for which hydration and parenteral feeding were undertaken). In order to exclude comorbid physical conditions, numerous tests were carried out, but no pathology worthy of note was identified except for a sinus tachycardia with an in-therapy insertion of a beta blocker (Bisoprolol 3.75 mg/day). Due to the persistence of the aforementioned symptoms, the patient was transferred to our facility according to previous agreements.

### 2.2. Acute Presentation

On admission to our ward the patient was inaccessible to interview and not collaborating with negativism. The patient was confused and disoriented in space and time, and towards things and people. The patient’s facial expression was hypomimic, and their gestures were hyper-represented with the presence of stereotyped and mannered movements. The speech was slowed down, which exhibited derailment and tangentiality. The tone of the mood, as well as the content of the thought, proved difficult to investigate. Spontaneous feeding was absent, so nutrition and pharmacological treatments were administered by nasogastric (NG) tubing. A urinary catheter and a peripherally inserted central catheter (PICC) in the left basilica vein, were also positioned.

### 2.3. Hospital Course

In accordance with the anamnesis, the described clinical picture, and the Bush–Francis Catatonia Rating Scale (BFCRS) [9], which scored 36, a diagnosis of a delirious mania episode was established and psychopharmacological therapy based on Lorazepam IV (8 mg/day) and Sodium Valproate IV (800 mg/day) was started, while Bisoprolol was maintained due to sinus tachycardia. She received subcutaneous Enoxaparin 4000 IU/day and enteral nutrition of 1000 mL of nutritional mixture with 1.5 kcal/mL through the NG tube. In the subsequent days, Lorazepam IV was increased up to 12 mg per day and the patient showed a mild response. During hospitalization fever and neutrophilic leucocytosis were detected, so a therapy based on intramuscular (IM) beta-lactam antibiotics (Ceftriaxon) was set up while a chest X-ray did not document “any significant parenchymal changes with an outbreak or effusion character”. Oxygen therapy was also set up for O2 desaturation episodes due to apneas. For the persistence of fever and leucocytosis, urgent blood tests were required to assess blood cell count, fibrinogen, and procalcitonin, whilst PICC and peripheral veins cultures, arterial blood gas analysis (ABG), and replacement of the urinary catheter and culture of the catheter tip were also completed. These examinations showed leucocytosis (11.5 × 10^3^/microL), increased neutrophil granulocytes (7.86 × 10^3^/microL), PCR 1.14 mg, PCT 0.07 ng/mL, and fibrinogen 492 mg/dL with mild respiratory alkalosis; blood cultures were negative while the catheter tip culture detected the presence of Enterococcus Faecalis and Corynebacterium Amycolatum. Therefore, Amoxicillin 1500 mg/day antibiotic therapy was administrated orally. Given the persistence of disorientation, stereotypies, negativism, and echolalia, it was decided to undertake a cycle of ECT. Cardiological and cerebral imaging examinations were required to exclude contraindications to the therapy. A head CT without contrast medium conducted under general anaesthesia, given the lack of reliability of previous neuroimaging tests, was unable to show “any findings that contraindicated electroconvulsive therapy”. We obtained informed consent from the legal guardian of the patient and started ECT. We employed psychopharmacological therapy with Sodium Valproate (300 mg/day) and Chlorpromazine (150 mg/day). Bilateral ECT was delivered using a brief pulse stimulator Mecta 5000Q on a twice-a-week schedule. Anaesthesia was induced with Thiopental IV (2–4 mg/kg) and muscle relaxation was assured with Succinylcholine IV (0.5–1 mg/kg). The stimulus dosage was initially based on the half-age method [19]. Motor and electroencephalogram (EEG) seizure duration were monitored. During the ECT course (eleven sessions) the stimulus dosage was adjusted to maintain a seizure duration of at least 25 s. If the seizure duration decreased to <25 s, the stimulus dosage was increased (to 1.5 times the previous setting) at the following session.

## 3. Results

After the sixth application, there was an improvement in motor symptomatology with the execution of voluntary movements and the absence of stereotypies with a recovery including walking, the reappearance of appropriate facial expression, and the resumption of oral autonomous feeding with the removal of the NG tube. Confusion, spatio-temporal disorientation, mnesic deficit, perplexity, and restlessness were resolved after eleven applications. At the discharge, after the last application, the patient appeared vigilant, alert, and oriented with mnesic shortcomings concerning the last months. The mood was stable, accompanied with good planning, and an increase in target activities. The BFCRS score was 3. The maintenance therapy was based on Lithium salts (300 mg/day), Sodium Valproate (750 mg/day), Chlorpromazine (100 mg/day), and Bisoprolol (3.75 mg/day).

## 4. Discussion

The picture of our patient met the criteria of the BFCRS [9] score and those for the catatonia of the DSM-5 [11] (Table 2).

The BFCRS is a standardized tool for establishing a diagnosis of catatonia and quantifying its severity. It consists of a 23-item rating scale with a score for each ranging from 0 to 3. The first 14 items represent the screening tool. The symptoms described here are considered absent if the score is equal to 0, or present if the score is equal to or greater than 1. The presence of at least two symptoms on this scale allows the hypothesis of catatonia to be made [9]. A total score above 30 indicates severe symptomatology, as confirmed by studies or other observations [20,21].

Among the catatonic symptoms, catalepsy, mutism, mannerisms, rigidity, waxy flexibility, stupor, staring, grimacing, negativism, stereotypy, withdrawal, ambitendency, perseveration, and autonomic abnormality were present. There was an alteration of the respiratory and heart rates. At initial observation, the patient had a score of 36 for BFCRS, the same dropout score was 3. The anamnesis and the clinical inspection revealed a rapid onset of confusion, disorientation, and psychomotor agitation, typical of delirium. However, it was not possible to prove, given the lack of access to the patient, if she had episodes of auditory or visual hallucinations or delusions during the hospitalization, described in the literature as part of the symptomatology of delirious mania.

These data, together with the clinical history prior to the onset of catatonic symptoms, characterized by a severe manic phase with mixed symptoms, made us orientate towards a form of catatonia, specifically delirious mania. This syndrome should be in differential diagnosis with other forms of catatonia, particularly neuroleptic malignant syndrome, also considering the numerous prescriptions of antipsychotic drugs. This was excluded because of the absence of hyperthermia. The fever presented by the patient during hospitalization was attributed to an infection, which resolved after administration of antibiotics. However, the distinction between the various forms of catatonia turns out to be less important in clinical practice than the treatment of the signs and symptoms of the individual case.

Prevention and monitoring of general medical conditions are the first step in the treatment of this syndrome. It is therefore important to ensure adequate water intake, nutrition, and proper diuresis and evacuation. Attention must be placed on aspiration pneumonia, pulmonary embolism, infections (particularly pulmonary, integumentary, and urinary), ileus, skin ulcerations, and rhabdomyolysis. In case of signs of malignancy, it is necessary to discontinue antipsychotic drugs and ensure intensive support and monitoring [13]. A urinary catheter and a NG tube were placed to ensure proper nutrient and fluid intake and urinary elimination. In order to avoid possible embolization given the position of the patient, Enoxaparin, once daily was included in the therapy. Bisoprolol was maintained for tachycardia. In response to signs and symptoms typical of pneumonia, intramuscular and oral antibiotics, cortisone, and oxygen therapy were administrated until resolution.

We have considered the case history of the patient to be of particular interest since there were no underlying medical causes, and no response was observed with administration of mood stabilizers and antipsychotics. The patient showed a response to the administration of BZD IV with partial improvement of the clinical picture, showing instead a complete response to treatment with ECT, which is considered for this type of framework to be the gold therapeutic standard [9,10]. The efficacy of BZDs in the treatment of catatonia in its various forms is based on the pathogenic hypotheses described earlier: these drugs, in fact, by enhancing GABAergic activity aim to restore the abnormality of its activity in the cortex and its possible down-stream effects on dopaminergic and glutamatergic function [13]. The effectiveness of ECT in catatonic states is widely recognized. It is a safe and effective treatment in these syndromes, particularly in association with mood disorders refractory to drug therapy, with a response rate ranging from 80% to 90% [22].

## 5. Conclusions

Delirious mania is frequently associated with catatonic symptoms. More than half of patients with catatonia have manic-type symptoms. In some cases, after an initial exacerbation of affective symptoms and confusion, catatonic symptoms occur as the disease progresses. These data show how important it is to diagnose this symptom’s structure as soon as possible to implement an effective therapy and thus avoid potentially fatal complications. We diagnosed a form of delirious mania associated with symptoms of malignancy, so the use of antipsychotics was avoided. An intensive care of life-threatening general medical conditions was setup and the patient was treated by the administration of BZD IV, and subsequently by ECT, which guaranteed us a good level of efficacy and a reduction of possible side effects from drugs, obtaining a complete resolution of the clinical picture.

## Figures and Tables

**Table 1:** The Catatonia classification. This table show the different forms of presentation of catatonia.

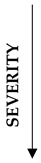	**Classification**	**Catatonia Form**
	Non-malignant form	Kahlbaum syndrome (Inhibited Catatonia)
	Intermediate forms	Excited Catatonia
		Delirious Mania
		Oneiroid state
	Malignant forms	Malignant Catatonia
		Neuroleptic malignant syndrome
		Serotonine syndrome

**Table 2:** Catatonia criteria. This tables summarizes all possible catatonic symptoms following the criteria of the Bush-Francis Catatonia Rating Scale (BFCRS) and the Diagnostic and Statistical Manual of Mental Disorders—Fifth Edition (DSM-5).

**BFCRS Criteria**	
1. Excitement	13. Waxy Flexibility
2. Immobility/stupor	14. Withdrawal
3. Mutism	15. Impulsivity
4. Staring	16. Automatic obedience
5. Posturing/catalepsy	17. Mitgehen
6. Grimacing	18. Gegenhalten
7. Echopraxia/echolalia	19. Ambitendency
8. Stereotipy	20. Grasp reflex
9. Mannerisms	21. Perseveration
10. Verbigeration	22. Combativeness
11. Rigidity	23. Autonomic abnormality
12. Negativism	
**DSM-5 Criteria**	
1. Stupor	7. Mannerism
2. Catalepsy	8. Stereotypy
3. Waxy flexibility	9. Agitation
4. Mutism	10. Grimacing
5. Negativism	11. Echolalia
6. Posturing	12. Echopraxia

## Data Availability

The data are not publicly available due to the regional laws restrictions.
